# Olfactory ensheathing cells abutting the embryonic olfactory bulb express *Frzb*, whose deletion disrupts olfactory axon targeting

**DOI:** 10.1002/glia.23515

**Published:** 2018-09-26

**Authors:** Constance A. Rich, Surangi N. Perera, Jacqueline Andratschke, C. Claus Stolt, Dennis P. Buehler, E. Michelle Southard‐Smith, Michael Wegner, Stefan Britsch, Clare V. H. Baker

**Affiliations:** ^1^ Department of Physiology, Development and Neuroscience University of Cambridge Cambridge United Kingdom; ^2^ Institute of Molecular and Cellular Anatomy Ulm University Ulm Germany; ^3^ Institut für Biochemie, Emil‐Fischer‐Zentrum Friedrich‐Alexander‐Universität Erlangen‐Nürnberg Erlangen Germany; ^4^ Division of Genetic Medicine, Department of Medicine Vanderbilt University School of Medicine Nashville Tennessee

**Keywords:** GnRH neurons, OECs, olfactory receptor neurons, Omp, Sox10

## Abstract

We and others previously showed that in mouse embryos lacking the transcription factor Sox10, olfactory ensheathing cell (OEC) differentiation is disrupted, resulting in defective olfactory axon targeting and fewer gonadotropin‐releasing hormone (GnRH) neurons entering the embryonic forebrain. The underlying mechanisms are unclear. Here, we report that OECs in the olfactory nerve layer express *Frzb*—encoding a secreted Wnt inhibitor with roles in axon targeting and basement membrane breakdown—from embryonic day (E)12.5, when GnRH neurons first enter the forebrain, until E16.5, the latest stage examined. The highest levels of *Frzb* expression are seen in OECs in the inner olfactory nerve layer, abutting the embryonic olfactory bulb. We find that *Sox10* is required for *Frzb* expression in OECs, suggesting that loss of Frzb could explain the olfactory axon targeting and/or GnRH neuron migration defects seen in *Sox10*‐null mice. At E16.5, *Frzb*‐null embryos show significant reductions in both the volume of the olfactory nerve layer expressing the maturation marker Omp and the number of Omp‐positive olfactory receptor neurons in the olfactory epithelium. As Omp upregulation correlates with synapse formation, this suggests that *Frzb* deletion indeed disrupts olfactory axon targeting. In contrast, GnRH neuron entry into the forebrain is not significantly affected. Hence, loss of Frzb may contribute to the olfactory axon targeting phenotype, but not the GnRH neuron phenotype, of *Sox10*‐null mice. Overall, our results suggest that Frzb secreted from OECs in the olfactory nerve layer is important for olfactory axon targeting.

## Main Points

- *Frzb* is expressed by olfactory ensheathing cells abutting the embryonic mouse olfactory bulb.
- *Frzb* expression requires *Sox10*. Deletion of *Frzb* disrupts olfactory receptor neuron maturation, likely reflecting a defect in olfactory axon targeting.

## 1 INTRODUCTION

During early olfactory nerve development, the “migratory mass” must forge its own route through the frontonasal mesenchyme, from the olfactory placode to the forebrain (Balmer & LaMantia, 2005). The migratory mass comprises olfactory axons (arising from olfactory receptor neurons in the olfactory epithelium), olfactory placode‐derived migratory neurons, and olfactory ensheathing glial cells (OECs) (Blanchart, Martín‐López, De Carlos, & López‐Mascaraque, 2011; Miller, Treloar, & Greer, 2010b; Valverde, Santacana, & Heredia, 1992). OECs, which are neural crest‐derived (Barraud et al., 2010), migrate ahead of olfactory axons during both development and regeneration (Chehrehasa et al., 2010; Tennent & Chuah, 1996; Valverde et al., 1992), while in explant culture, olfactory axons prefer to grow on OECs and extend only in association with migrating OECs (Windus et al., 2011). Hence, OECs may facilitate the migration of olfactory axons and migrating neurons to the forebrain. Once the migratory mass reaches the forebrain, olfactory axon fascicles ensheathed by OECs form the presumptive olfactory nerve layer (ONL) that caps the developing olfactory bulb (Treloar, Miller, Ray, & Greer, 2010; Valverde et al., 1992). As the basement membrane around the olfactory bulb breaks down, OEC processes and olfactory axons enter the main body of the bulb, and eventually olfactory axons synapse with the dendrites of target mitral/tufted neurons, forming the glomerular layer (Marin‐Padilla & Amieva, 1989; Valverde et al., 1992). Olfactory axons remain in the outer ONL until they are near their glomerular target, at which point they enter the inner ONL and “sort out” into mostly homotypic axon bundles (i.e., bundles comprising mostly olfactory axons expressing the same odorant receptor), before converging on the target glomerulus (Akins & Greer, 2006a; Miller, Maurer, Zou, Firestein, & Greer, 2010a; Treloar et al., 2010; Treloar, Feinstein, Mombaerts, & Greer, 2002; Treloar, Purcell, & Greer, 1999).

Our lab and others recently found that defects in OEC differentiation, caused by genetic deletion of the transcription factor Sox10 (which is expressed within the embryonic olfactory system only by developing OECs and, much later in development, by mucus‐secreting Bowman's gland cells within the olfactory epithelium; Barraud et al., 2010; Forni, Taylor‐Burds, Melvin, Williams, & Wray, 2011; Barraud, St John, Stolt, Wegner, & Baker, 2013; Pingault et al., 2013), disrupt both olfactory axon targeting and the entry of migrating gonadotropin‐releasing hormone (GnRH) neurons into the embryonic mouse forebrain (Amaya et al., 2015; Barraud et al., 2013; Pingault et al., 2013). GnRH neurons migrate from the vomeronasal organ (a specialized region of the olfactory epithelium) along caudally projecting vomeronasal and terminal nerve axons—which are also ensheathed by OECs—to enter the ventromedial forebrain, eventually reaching the hypothalamus, where they are required for gonadotropin release at puberty (Cariboni, Maggi, & Parnavelas, 2007; Schwanzel‐Fukuda & Pfaff, 1989; Taroc, Prasad, Lin, & Forni, 2017; Wray, 2010; Wray, Grant, & Gainer, 1989; Yoshida, Tobet, Crandall, Jimenez, & Schwarting, 1995). Migrating GnRH neurons, and other neurons within the migratory mass, are intimately associated with OECs (Geller, Kolasa, Tillet, Duittoz, & Vaudin, 2013; Miller et al., 2010b). In *Sox10*‐null mice, the expression of glial markers is significantly reduced in the neural crest‐derived cells that persist on the olfactory nerve and within the ONL (Amaya et al., 2015; Barraud et al., 2013; Pingault et al., 2013); olfactory axons accumulate ventromedial to the olfactory bulbs and the ONL is thinner, suggesting an olfactory axon targeting defect (Amaya et al., 2015; Barraud et al., 2013). Significantly fewer olfactory receptor neurons in the epithelium express olfactory marker protein (Omp) at E16.5 (Barraud et al., 2013), most likely owing to the axon targeting defect, given that Omp is a maturation marker whose expression correlates with the onset of synaptogenesis (Farbman & Margolis, 1980; Monti Graziadei, Stanley, & Graziadei, 1980). The failure of normal OEC differentiation in *Sox10*‐null mouse embryos also disrupts the entry into the embryonic forebrain of GnRH neurons (Barraud et al., 2013; Pingault et al., 2013). These data explain why loss‐of‐function mutations in human *SOX10* have been found to underlie some cases of Kallmann's syndrome, that is, combined anosmia and sterility (hypogonadotropic hypogonadism) (Pingault et al., 2013), but the underlying mechanisms are unknown.

During a survey of Wnt signaling pathway gene expression during OEC development (CAR and CVHB, unpublished), we were intrigued to see expression of *Frzb* (*secreted frizzled‐related protein 3*, *Sfrp3*) in a subset of OECs at the embryonic olfactory nerve/forebrain junction. *Frzb* expression was previously reported in the olfactory nerve layer in postnatal and young adult mice (Shimogori, VanSant, Paik, & Grove, 2004). Sfrp family members inhibit both “canonical” and “noncanonical” Wnt pathways, but also have Wnt‐independent activities, including in axon extension and guidance (Cruciat & Niehrs, 2013). *Frzb* itself is expressed by neurons in the dorsal horn of the spinal cord, and its deletion disrupts innervation of the dorsal horn by cutaneous afferents (John et al., 2012). Furthermore, during primary mouth formation in *Xenopus*, the basement membrane persists after Frzb knockdown (Dickinson & Sive, 2009). Taken together, this suggested to us that Frzb secreted from OECs bordering the embryonic olfactory bulb could be involved in olfactory axon targeting and/or basement membrane breakdown around the developing olfactory bulb, which could be important for GnRH neuron entry. Loss of Frzb could therefore explain, at least in part, the defects in olfactory axon targeting and GnRH neuron entry into the forebrain seen in *Sox10*‐null mice, in which normal OEC differentiation is disrupted (Amaya et al., 2015; Barraud et al., 2013; Pingault et al., 2013).

Here, we report the time‐course of *Frzb* expression and demonstrate that *Frzb* is downstream of Sox10 in OECs. We show that the volume of the Omp‐immunoreactive ONL of *Frzb*‐null mouse embryos is significantly reduced, and that there are fewer mature (Omp‐positive) olfactory receptor neurons in the olfactory epithelium. As Omp expression correlates with synapse formation (Farbman & Margolis, 1980; Monti Graziadei et al., 1980), this suggests that loss of Frzb indeed results in defective olfactory axon targeting. However, the proportion of GnRH neurons entering the forebrain is not significantly affected. Overall, our data suggest that Frzb secretion from a subpopulation of OECs abutting the embryonic olfactory bulb plays a role in olfactory axon targeting.

## 2 MATERIALS AND METHODS

### 2.1 Mouse embryos

*Frzb* knockout and *Sox10* ^*lacZ*^ mutant mice were previously described (Britsch et al., 2001; Lories et al., 2007). All mouse experiments were carried out in accordance with German law and were approved by the respective governmental offices in Ulm and Erlangen, and, for a subset of *Sox10* ^*lacZ*^ mutant embryos analyzed, by the Vanderbilt University Institutional Animal Care and Use Committee. Litters from timed pregnancies were harvested at various days *post coitus*, counting the day of plug detection as embryonic day (E)0.5. Genotyping of tail biopsies was performed by PCR as previously described (Britsch et al., 2001; Lories et al., 2007).

### 2.2 Cryosectioning and in situ hybridization

Embryos were immersion‐fixed in 4% paraformaldehyde in phosphate‐buffered saline (PBS) overnight at 4°C, then embedded in OCT (Thermo Fisher Scientific) for cryosectioning at 10 μm. In situ hybridization was performed on sections as previously described (Miller, Perera, & Baker, 2017). The mouse *Frzb* clone was a gift of Christine Hartmann (Westfälische Wilhelms‐Universität Münster, Münster, Germany). A 405‐bp fragment of mouse *Npy* cDNA, corresponding to base‐pairs 86–490 of NCBI reference sequence NM_023456.3, was PCR‐amplified (forward primer CGCCACGATGCTAGGTAACAA; reverse primer CTAGTGGTGGCATGCATTGGT) from single‐strand cDNA (prepared using Thermo Fisher's High‐Capacity cDNA Reverse Transcription Kit on total RNA extracted with Trizol [Invitrogen] from E13.5 mouse embryo heads). The *Npy* cDNA fragment was cloned into pDrive (Qiagen) using the Qiagen PCR cloning kit and sequenced (Biochemistry Department DNA Sequencing Facility, University of Cambridge, UK). Primer‐BLAST software from NCBI (Ye et al., 2012) was used to design PCR primers and check their specificity. Primer melting temperature and self‐complementarity were checked using Primer3Plus (https://primer3plus.com/cgi-bin/dev/primer3plus.cgi; Untergasser et al., 2012). Digoxigenin‐labeled antisense riboprobes were generated as described (Henrique et al., 1995).

### 2.3 Immunohistochemistry

Immunohistochemistry was performed as previously described (Miller et al., 2017), except that heat‐inactivated sheep, goat, or donkey serum, as appropriate, was used at 10% in primary and secondary antibody solutions for blocking. When necessary, antigen retrieval was performed by heating the slides for 4 min (until boiling) in a microwave in 10 mM sodium citrate buffer solution (pH 6), followed by two washes in PBS. Primary antibodies were used against the following antigens: β‐galactosidase (chicken, Abcam ab9361; 1:1000), FABP7 (fatty acid binding protein 7, an early glial marker also known as brain lipid‐binding protein, BLBP; rabbit, Millipore ABN14; 1:150), gonadotropin‐releasing hormone (GnRH; rabbit, Abcam ab5617; 1:150), olfactory marker protein (Omp, expressed by mature olfactory receptor neurons; goat, Wako 019‐22291; 1:500), peripherin (a neuron‐specific intermediate filament protein; rabbit, Millipore; 1:200), Sox10 (rabbit, gift of Vivian Lee, Medical College of Wisconsin, WI, 1:3000; Meng, Yuan, & Lee, 2011; Yardley & García‐Castro, 2012), Tubb3 (neuronal βIII tubulin; mouse IgG2a, clone TUJ1, Covance MMS‐435P; 1:250). Appropriately matched Alexa Fluor‐conjugated secondary antibodies (Molecular Probes) were used at 1:1000. For triple immunostaining, anti‐Tubb3 was detected by incubating with a biotinylated secondary antibody (goat anti‐mouse IgG2a, Invitrogen, 1:100; horse anti‐mouse IgG, Vector Laboratories, 1:200), followed by Alexa350‐conjugated NeutrAvidin (Molecular Probes, 1:100).

### 2.4 Analysis of olfactory nerve layer volume

Heterozygous *Frzb* ^*+/−*^ mutant embryos and *Frzb*‐null embryos at E16.5 were serially cryosectioned at 10 μm (10 slides/series: on each slide, each section was collected every 100 μm), in either the coronal or parasagittal plane, and slides immunostained for the mature olfactory axon marker Omp (Farbman & Margolis, 1980; Monti Graziadei et al., 1980), and the general axon marker Tubb3 (neuronal βIII tubulin). Coronal series spanned the rostrocaudal extent of the olfactory bulbs; parasagittal series spanned the width of the forebrain (i.e., both olfactory bulbs). Low‐power (10×) images were captured of each section on one slide for each series containing olfactory bulb from each embryo (or occasionally sections from two slides, if the region containing the olfactory bulbs spanned more than one 10‐slide series). The freehand selection and measure tools in NIH ImageJ software (Schneider, Rasband, & Eliceiri, 2012) were used to draw around and measure the area of Omp expression (i.e., the area of the olfactory nerve layer) in μm^2^ for each 10× image with Omp immunostaining. For each of 4 heterozygous *Frzb* ^*+/−*^ embryos and 5 homozygous *Frzb* ^*−/−*^ embryos, these areas were summed and multiplied by the 10 μm thickness of the sections to give an estimate of one‐tenth of the total volume of the Omp‐positive olfactory nerve layer (across both olfactory bulbs) per embryo.

### 2.5 Analysis of olfactory receptor neuron maturation and olfactory epithelium thickness

Confocal images were taken of 10 μm serial sections in either the coronal or parasagittal plane (10 slides/series: on each slide, each section was collected every 100 μm), immunostained for the maturation marker Omp (Farbman & Margolis, 1980; Monti Graziadei et al., 1980) and the general axonal/neuronal marker Tubb3, from four heterozygous *Frzb* ^*+/−*^ embryos and four *Frzb*‐null embryos at E16.5. On coronal sections, Adobe Photoshop CS6 was used to place a 200 μm bar in the image (in the Tubb3 channel, to reduce bias) along the dorsal olfactory epithelium on left and right sides of the nasal septum (i.e., 2 measurements per section), for 3–4 sections per embryo (i.e., 6–8 measurements per embryo). Similarly, on parasagittal sections, a bar was placed in the image along the dorsal olfactory epithelium, for 6 sections per embryo (i.e., 6 measurements per embryo). Within the region of olfactory epithelium selected by the bar, all Tubb3‐positive cells (i.e., neurons) and all double Omp‐positive, Tubb3‐positive cells (i.e., mature neurons) were counted, and the thickness of the epithelium measured at 3 points (at each edge of the bar, and at a third point near the center of the bar).

### 2.6 Analysis of GnRH neuron numbers

GnRH neurons (identified as GnRH‐immunoreactive, Tubb3‐positive cells) were counted on 10 μm sections through the forebrain in either the coronal or parasagittal plane (10 slides/series: on each slide, each section was collected every 100 μm) of three E16.5 heterozygous *Frzb* ^*+/−*^ embryos and three E16.5 *Frzb*‐null embryos.

### 2.7 Statistical analysis

Microsoft Excel was used for initial data analysis. GraphPad Prism 7 (GraphPad Software, La Jolla, CA) was used to generate scatter plots showing mean and standard deviation (*SD*), to test all datasets for normality using the Shapiro–Wilk test, and to compare variances using an *F* test. For datasets passing the Shapiro–Wilk normality test (*p* > .05), means were compared in GraphPad Prism 7 using an unpaired two‐tailed Student's *t* test. For datasets where *p* < .05 for the Shapiro–Wilk normality test, means were compared in GraphPad Prism 7 using the Mann–Whitney (Wilcoxon rank sum) test.

### 2.8 Image capture and processing

Images were captured using either a Zeiss AxioSkop 2 MOT compound microscope with a QImaging Retiga 2000R camera and an RGB pancake (QImaging), using QCapture Pro 6.0 software, or a Nikon A1 confocal with a Photometrics Evolve EM‐CCD‐camera running on Nikon elements software. Adobe Photoshop CS6 was used to process images.

## 3 RESULTS

### 3.1 *Frzb* is expressed by central OECs from E12.5 until at least E16.5, with strongest expression in the inner olfactory nerve layer, next to the olfactory bulb

**Figure 1:**
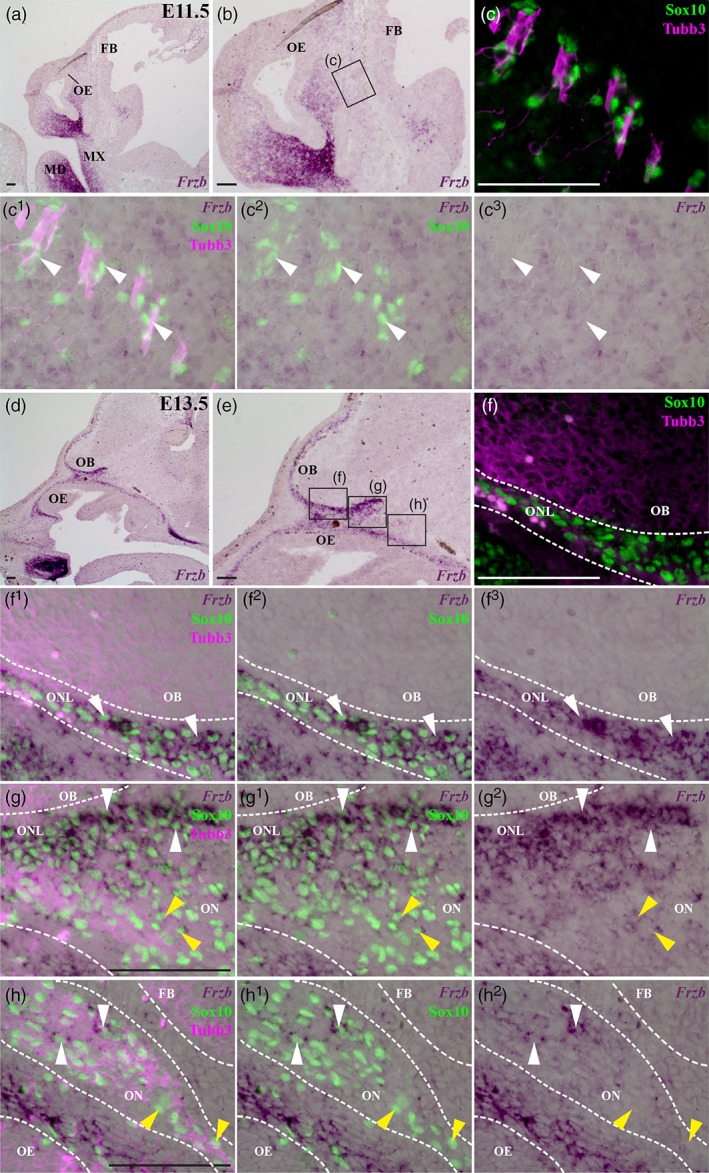
*Frzb* is expressed by OECs in the embryonic mouse olfactory nerve layer. In situ hybridization for *Frzb* on parasagittal sections through the developing mouse olfactory system. (a–c^3^) At E11.5, *Frzb* expression is seen in the maxillary and mandibular prominences, and in frontonasal mesenchyme caudal to the olfactory epithelium, but not in OECs (identified by nuclear immunoreactivity for the transcription factor Sox10) associated with Tubb3‐positive olfactory nerve fascicles (arrowheads highlight examples). (d–h^2^) At E13.5, strong *Frzb* expression can be seen in OECs in the olfactory nerve layer adjacent to the olfactory bulb (white arrowheads, f^1^–g^2^), but in fewer OECs on the olfactory nerve more peripherally (yellow arrowheads, g–g^2^; white arrowheads, h–h^2^). Yellow arrowheads in panels h–h^2^ highlight examples of Frzb‐negative peripheral OECs. FB, forebrain; MD, mandibular prominence; MX, maxillary prominence; OB, olfactory bulb; OE, olfactory epithelium; ON, olfactory nerve; ONL, olfactory nerve layer. Scale bar: 100 μm [Color figure can be viewed at wileyonlinelibrary.com]

At embryonic day (E)10.5 in the mouse, the “migratory mass” of olfactory receptor neuron axons, migrating neurons and *Sox10*‐expressing OEC precursors has formed subjacent to the invaginated olfactory epithelium (Barraud et al., 2013; Forni et al., 2011; Miller et al., 2010b; Valverde et al., 1992). In situ hybridization on parasagittal sections of wild‐type embryos at E10.5 revealed *Frzb* expression in mesenchyme in the maxillary and mandibular prominences, but not in the developing olfactory system (*n* = 2; data not shown). By E11, developing OECs can be identified by Sox10 expression (Barraud et al., 2013; Forni et al., 2011) or by immunoreactivity for the early glial marker fatty acid binding protein 7 (Fabp7; also known as brain fatty acid‐binding protein, Bfabp, or brain lipid‐binding protein, Blbp) (Miller et al., 2010b), and their processes wrap both olfactory axons and migrating neurons in the migratory mass (Miller et al., 2010b). The migratory mass extends along the rostromedial surface of the telencephalon without contacting it until E11.5, when olfactory axons contact the future olfactory bulb region at the rostral tip of the telencephalon, but without penetrating the basal membrane (Doucette, 1989; Marin‐Padilla & Amieva, 1989; Treloar et al., 2010). At E11.5 in wild‐type embryos, *Frzb* expression was detected in maxillary and mandibular mesenchyme, and also in frontonasal mesenchyme caudal to the olfactory epithelium, but not in OECs, which were identified by immunoreactivity for Fabp7 or Sox10, in association with olfactory nerve fascicles (*n* = 6; Figure 1a–c^3^ shows an embryo in which OECs were identified by Sox10 immunoreactivity).

At E12, a few “pioneer” olfactory axons penetrate the basal membrane of the forebrain via small fenestrations, while by E12.5, the olfactory bulb is morphologically distinguishable as a telencephalic evagination, capped by a thin presumptive olfactory nerve layer (ONL) (Doucette, 1989; Marin‐Padilla & Amieva, 1989; Treloar et al., 2010). Also at E12.5, the first GnRH neurons enter the forebrain, migrating along a caudal branch of the vomeronasal nerve and terminal nerve axons, which run medial to the olfactory nerve and are also ensheathed by OECs (Cariboni et al., 2007; Schwanzel‐Fukuda & Pfaff, 1989; Taroc et al., 2017; Wray et al., 1989; Yoshida et al., 1995). At E12.5–13.5, *Frzb* is expressed by a subset of OECs (identified by Sox10 or Fabp7 immunoreactivity in wild‐type embryos, or by β‐galactosidase‐immunoreactivity in heterozygous *Sox10* ^*lacZ/+*^ embryos, in which one allele of *Sox10* has been replaced by *lacZ*; Britsch et al., 2001; Barraud et al., 2013) in the olfactory nerve layer adjacent to the developing olfactory bulb, but in only a few cells more peripherally in the olfactory nerve (*n* = 10; Figure 1d–h^2^ shows an E13.5 embryo in which OECs were identified by Sox10 immunoreactivity).

**Figure 2:**
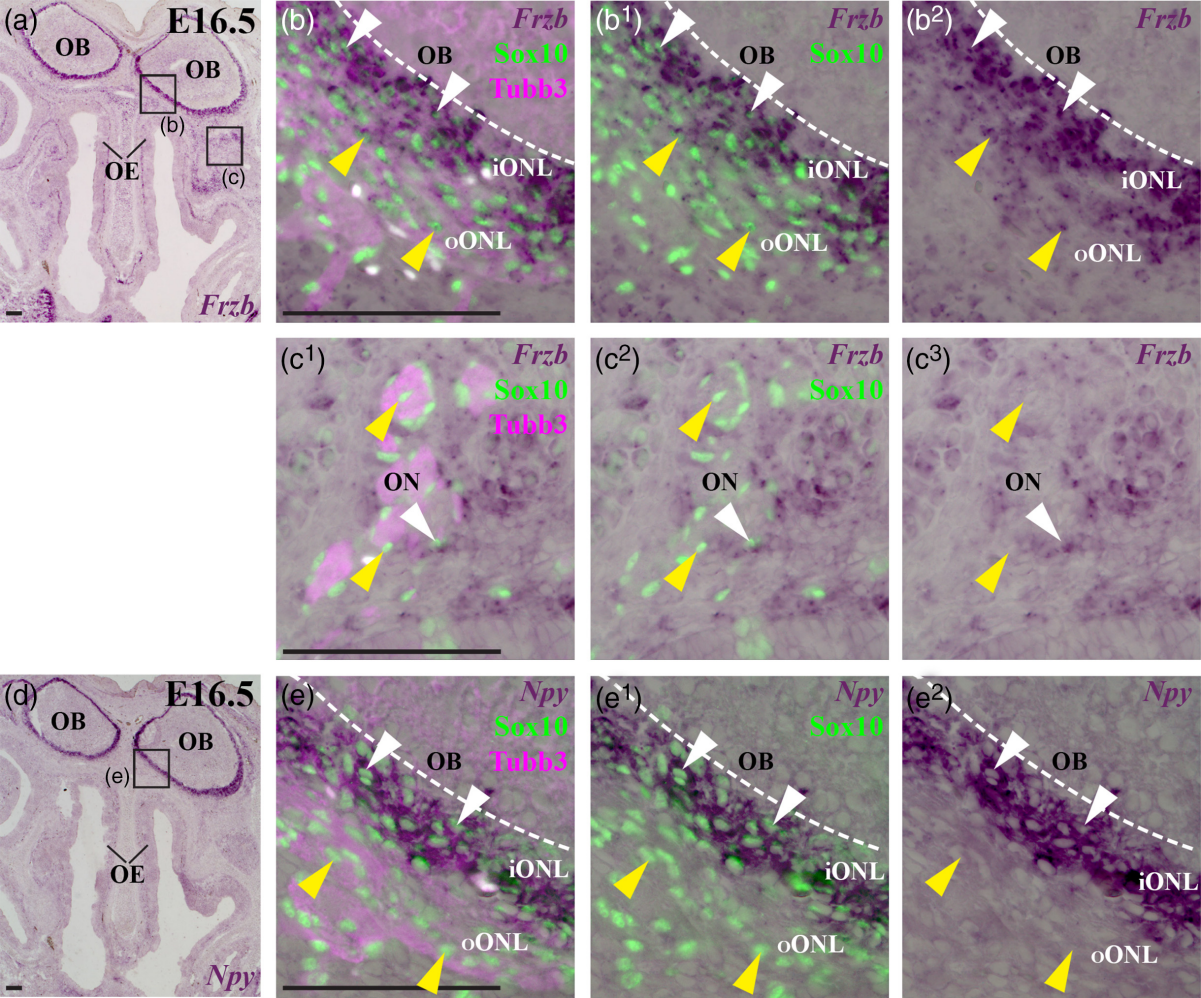
At E16.5, *Frzb* is expressed most strongly by OECs in the inner olfactory nerve layer. In situ hybridization for *Frzb* on parasagittal sections through the mouse olfactory system at E16.5. (a–b^2^) *Frzb* expression is seen in OECs (identified by nuclear Sox10 immunoreactivity) throughout the olfactory nerve layer, but more strongly in the inner region, adjacent to the olfactory bulb (white arrowheads) than in the outer region (yellow arrowheads). (c–c^2^) Few OECs associated with peripheral olfactory nerve fascicles on the same section seem to express *Frzb* (white arrowhead highlights an example): most are *Frzb*‐negative (yellow arrowheads highlight examples). (d–e^2^) In situ hybridization for the inner ONL‐specific OEC marker *Npy* on the adjacent serial section to the section shown in panels a–c^2^. iONL, inner olfactory nerve layer; OB, olfactory bulb; OE, olfactory epithelium; ON, olfactory nerve, ONL, olfactory nerve layer; oONL, outer olfactory nerve layer. Scale bar: 100 μm [Color figure can be viewed at wileyonlinelibrary.com]

From E13.5 to E16.5, the ONL thickens as more olfactory axons extend to the olfactory bulb and the basement membrane around the olfactory bulb continues to break down, but most olfactory axons remain restricted to the ONL until E15, when significant numbers of synapses are first seen (only a few synapses form earlier, at E14) and glomeruli start to form (Blanchart, De Carlos, & López‐Mascaraque, 2006; Hinds & Hinds, 1976a; Hinds & Hinds, 1976b; Marin‐Padilla & Amieva, 1989; Treloar et al., 2010). This “waiting period” has been suggested to enable olfactory axons expressing different odorant receptors to sort into homotypic fascicles before converging to form specific glomeruli (Treloar et al., 1999; Treloar et al., 2010): the sorting occurs only in the inner ONL (Miller et al., 2010a; Treloar et al., 2002). At the latest stage examined, E16.5 (*n* = 7), *Frzb* continues to be expressed by OECs in the ONL, most strongly in those OECs closest to the olfactory bulb (Figure 2a–b^2^), but in only a few peripheral OECs (Figure 2c–c^2^). OECs were identified as Sox10‐ or Fabp7‐immunoreactive cells in wild‐type embryos, or β‐galactosidase‐immunoreactive cells associated with the olfactory nerve in heterozygous *Sox10* ^*lacZ/+*^ embryos; in the embryo shown in Figure 2, OECs were identified by Sox10 immunoreactivity. In situ hybridization for the inner ONL‐specific OEC marker neuropeptide tyrosine (Npy) (Au, Treloar, & Greer, 2002; Ubink & Hokfelt, 2000) on alternate serial sections (Figure 2d–e^2^) confirmed that the strongest expression of *Frzb* in the ONL was in OECs in the inner ONL (compare Figure 2b–b^2^ with Figure 2e–e^2^).

Both GnRH neuron entry into the forebrain and olfactory axon targeting are disrupted in *Sox10*‐null embryos, in which OEC differentiation is defective (Barraud et al., 2013; Pingault et al., 2013), including loss of the inner ONL‐specific OEC marker Npy (Barraud et al., 2013). Our data show that *Frzb* is expressed by a subpopulation of OECs abutting the olfactory bulb, beginning from E12.5 (when the first GnRH neurons enter the developing bulb) and continuing through to at least E16.5. Hence, the loss of Frzb secretion from OECs adjacent to the olfactory bulb could be one of the molecular mechanisms underlying the defects in GnRH neuron entry into the forebrain and olfactory axon targeting that are seen in *Sox10*‐null embryos (Barraud et al., 2013; Pingault et al., 2013).

### 3.2 *Frzb* is downstream of *Sox10* in OECs

**Figure 3:**
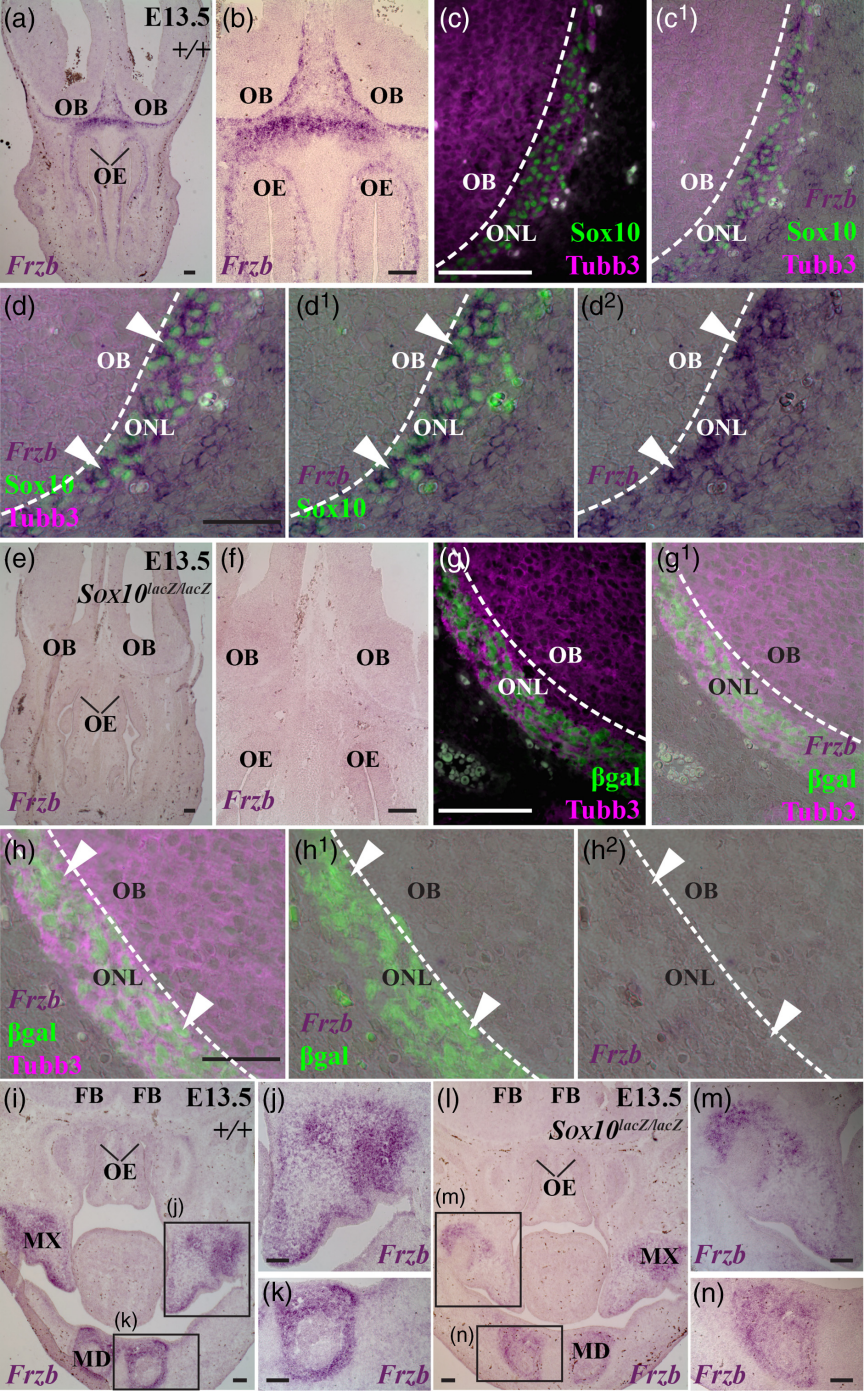
*Frzb* expression in the olfactory nerve layer depends on *Sox10*. Coronal sections through the developing olfactory system at E13.5 in *Sox10* ^*lacZ*^ mouse embryos, in which *lacZ* replaces one or both alleles of *Sox10* (Britsch et al., 2001). (a–d^2^) In a wild‐type embryo, *Frzb* expression can be seen in OECs (identified by immunoreactivity for nuclear Sox10) in the Tubb3‐positive olfactory nerve layer (arrowheads, d–d^2^). (e–h^2^) In a *Sox10* ^*lacZ/lacZ*^ (i.e., *Sox10*‐null) litter‐mate, *Frzb* expression is lacking in the olfactory nerve layer, where β‐galactosidase‐positive neural crest‐derived cells persist in the absence of Sox10 (Barraud et al., 2013) (arrowheads, h–h^2^). (i–k) In a more caudal section of the wild‐type embryo in panels a–d^2^ (from a different slide in the same round of in situ hybridization), *Frzb* expression is seen in mesenchyme in the maxillary and mandibular prominences. (l–n) In a more caudal section of the *Sox10* ^*lacZ/lacZ*^ (i.e., *Sox10*‐null) litter‐mate in panels e–h^2^ (from a different slide in the same round of in situ hybridization), *Frzb* expression is seen in mesenchyme in the maxillary and mandibular prominences. FB, forebrain; MD, mandibular prominence; MX, maxillary prominence; OB, olfactory bulb; OE, olfactory epithelium; ON, olfactory nerve. Scale bar: 100 μm [Color figure can be viewed at wileyonlinelibrary.com]

We first tested whether *Frzb* expression in developing OECs depends on Sox10, by comparing *Frzb* expression in wild‐type or heterozygous *Sox10* ^*lacZ/+*^ embryos versus *Sox10* ^*lacZ/lacZ*^ (*Sox10*‐null) littermates (Barraud et al., 2013; Britsch et al., 2001; Pingault et al., 2013). At E12.5‐E14.5, *Frzb* was expressed by OECs in the ONL in wild‐type and heterozygous *Sox10* ^*lacZ/+*^ embryos (*n* = 7; Figure 3a–d^2^), but was not detectable in the ONL in *Sox10*‐null littermates (*n* = 5; Figure 3e–h^2^). *Frzb* expression was seen in jaw mesenchyme for all genotypes (Figure 3i–n). Hence, *Frzb* expression in developing OECs requires *Sox10*. Furthermore, this is consistent with the hypothesis that the lack of Frzb secretion from OECs abutting the olfactory bulb may contribute to the disruption in *Sox10*‐null embryos of olfactory axon targeting and/or GnRH neuron entry into the forebrain (Barraud et al., 2013; Pingault et al., 2013). *Frzb*‐null mice are viable and fertile, but only 12% of the normal GnRH neuron population is required for fertility in males, and 12%–34% in females (Herbison, Porteous, Pape, Mora, & Hurst, 2008). So, it was still feasible that loss of Frzb could contribute to the phenotype of *Sox10*‐null embryos (Barraud et al., 2013; Pingault et al., 2013). We tested this hypothesis by comparing the development of the olfactory system in heterozygous *Frzb* ^*+/−*^ versus *Frzb*‐null mouse embryos (Lories et al., 2007).

### 3.3 The Omp‐immunoreactive olfactory nerve layer is thinner in *Frzb*‐null mice

**Figure 4:**
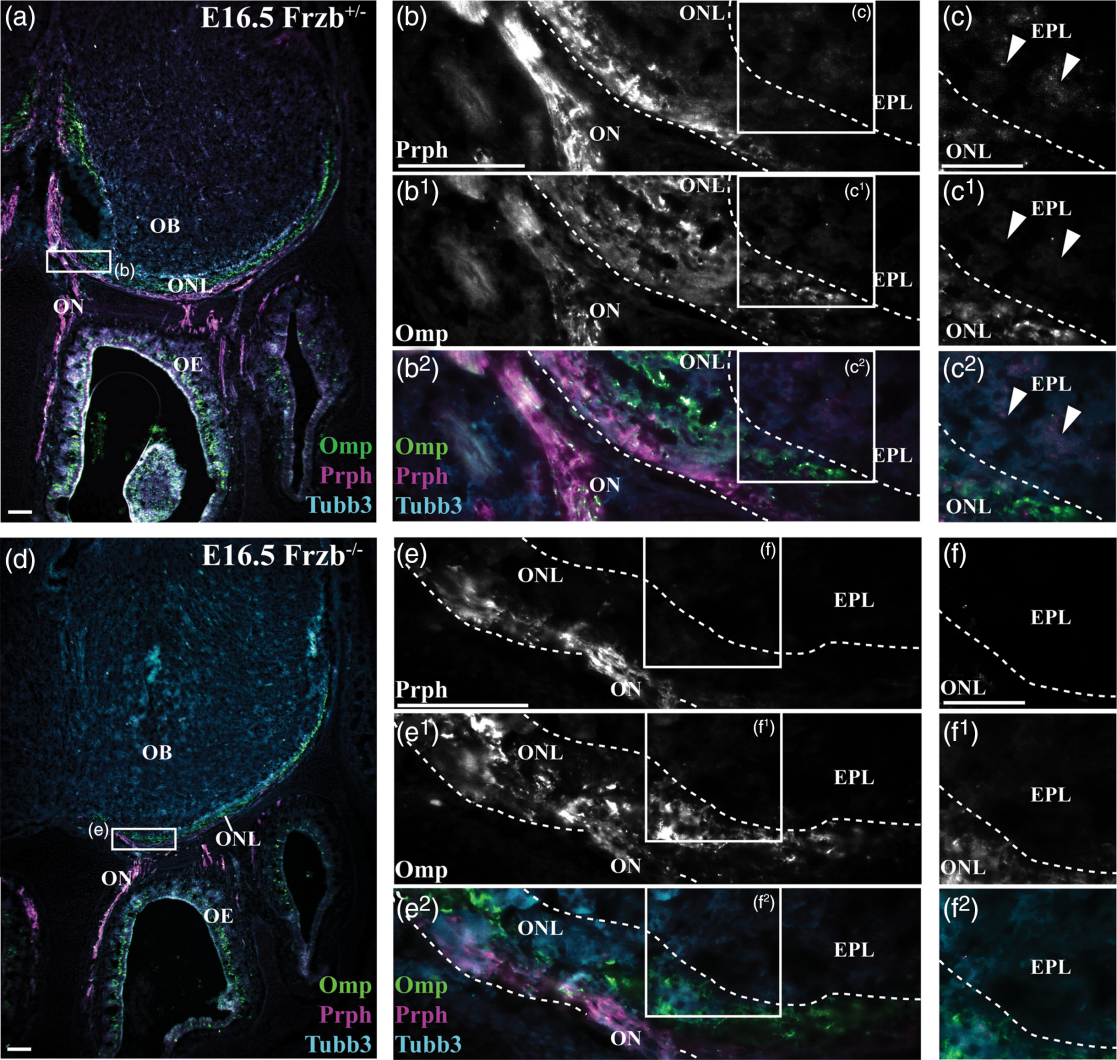
*Frzb* deletion seems to affect development of the olfactory nerve layer. Coronal sections through the mouse olfactory system at E16.5, immunostained for the maturation marker Omp, the outer ONL marker peripherin (Prph), and the general axonal marker Tubb3. (a–c^2^) In a heterozygous *Frzb* ^*+/−*^ embryo, Omp immunoreactivity labels the olfactory nerve, the full width of the ONL, and many olfactory receptor neurons in the olfactory epithelium, while peripherin immunoreactivity labels the olfactory nerve and the outer ONL. Some peripherin‐positive, Omp‐negative axons are also seen deep to the ONL, in the external plexiform layer (arrowheads, c–c^2^). (d–f^2^) In a *Frzb*‐null embryo, the Omp‐immunoreactive ONL seems to be thinner, and peripherin immunoreactivity is not detectable in the external plexiform layer. EPL, external plexiform layer; OB, olfactory bulb; OE, olfactory epithelium; ON, olfactory nerve; ONL, olfactory nerve layer. Scale bar: 50 μm (a,b,d,e) and 25 μm (c,f) [Color figure can be viewed at wileyonlinelibrary.com]

At E16.5, the entire width of the ONL can be visualized by immunoreactivity for the maturation marker Omp in heterozygous *Frzb* ^*+/−*^ mouse embryos (Figure 4a–c^2^), while the outer ONL can be identified by immunoreactivity for the type III neuron‐specific intermediate filament protein peripherin (Figure 4a–b^2^), as reported for wild‐type neonatal mice (Akins & Greer, 2006b). Peripherin‐positive, Omp‐negative axons can also be detected deep to the ONL on these coronal sections, in the external plexiform layer (Figure 4c–c^2^). (The proto‐glomerular layer is not well defined at this stage and is hard to distinguish from the ONL; Blanchart et al., 2006.) These could be “over‐shooting” olfactory axons, which in wild‐type rat and mouse embryos penetrate into deeper layers of the olfactory bulb, where they are pruned by radial glia (Amaya et al., 2015; Santacana, Heredia, & Valverde, 1992). This over‐shooting continues into the neonatal and postnatal period (Tenne‐Brown & Key, 1999; St John & Key, 2005).

**Figure 5:**
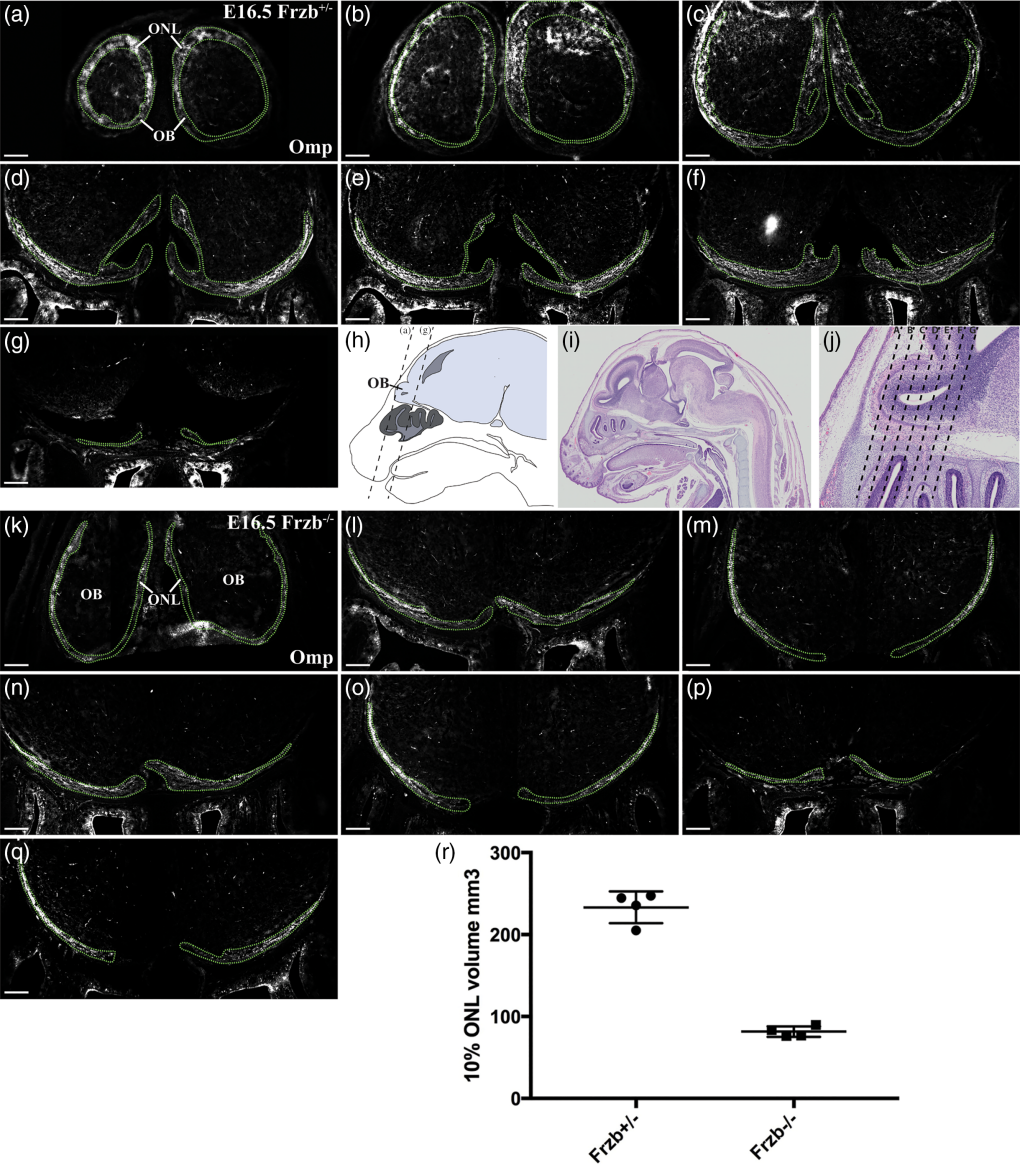
The volume of the Omp‐immunoreactive olfactory nerve layer is reduced after *Frzb* deletion. (a–g) Coronal sections moving rostrally to caudally through the olfactory bulbs of a heterozygous *Frzb* ^*+/−*^ mouse embryo, immunostained for Omp, with the Omp‐positive regions outlined. (h) Schematic showing a parasagittal view through an E16.5 mouse head, with dotted lines showing the approximate location of the most rostral and most caudal sections shown in (a–g). (i,j) Low‐power (i) and high‐power (j) views of a hematoxylin and eosin‐stained parasagittal section through an E16.5 mouse head (Plate 38c, image a, from the eHistology Atlas with Kaufman annotations; Graham et al., 2015). The dotted lines in (j) show the approximate locations of the sections shown in (a–g). (k–q) Coronal sections moving rostrally to caudally through the olfactory bulbs of a *Frzb*‐null mouse embryo, immunostained for Omp, with the Omp‐positive regions outlined. (r) Scatter plot showing the mean volume of one‐tenth of the Omp‐positive ONL at E16.5 in heterozygous *Frzb* ^*+/−*^ embryos (*n* = 4) and *Frzb*‐null embryos (*n* = 5). Error bars show *SD*. Scale bar: 200 μm [Color figure can be viewed at wileyonlinelibrary.com]

On coronal sections of *Frzb*‐null embryos at E16.5 (Figure 4d–f^2^), the Omp‐immunoreactive ONL generally seemed to be thinner than in heterozygous *Frzb* ^*+/−*^ embryos (compare Figure 4a with Figure 4d). Peripherin‐positive, Omp‐negative axons were not apparent deep to the ONL, suggesting that the loss of Frzb prevented olfactory axons from overshooting beyond the ONL. To quantify the apparent difference in ONL thickness, as defined by Omp immunoreactivity, we used ImageJ to outline all Omp‐positive regions on series of coronal or parasagittal sections through both olfactory bulbs at E16.5 (examples of coronal sections shown in Figure 5a–q), and from this calculated one‐tenth of the total volume of the Omp‐positive ONL (from both olfactory bulbs) per embryo. The mean ± standard deviation (*SD*) for this value was 233.20 mm^3^ ± 19.35 for heterozygous *Frzb* ^*+/−*^ embryos (*n* = 4) versus 85.17 mm^3^ ± 9.77 for *Frzb*‐null embryos (*n* = 5) (Figure 5r). Comparison of the means using an unpaired two‐tailed Student's *t* test showed that they were highly significantly different (*p* < .0001).

These data suggest that in the absence of Frzb secreted from the subpopulation of OECs abutting the olfactory bulb, there are fewer Omp‐positive (mature) axons in the ONL. As Omp expression correlates with the onset of synaptogenesis (Farbman & Margolis, 1980; Monti Graziadei et al., 1980), and can be upregulated in olfactory receptor neurons in embryonic olfactory epithelium co‐cultured in direct contact with the presumptive olfactory bulb (Chuah & Farbman, 1983), these results suggest that *Frzb* deletion disrupts olfactory axon targeting and thus, in consequence, the maturation of olfactory receptor neurons.

### 3.4 *Frzb* deletion disrupts olfactory receptor neuron maturation

**Figure 6:**
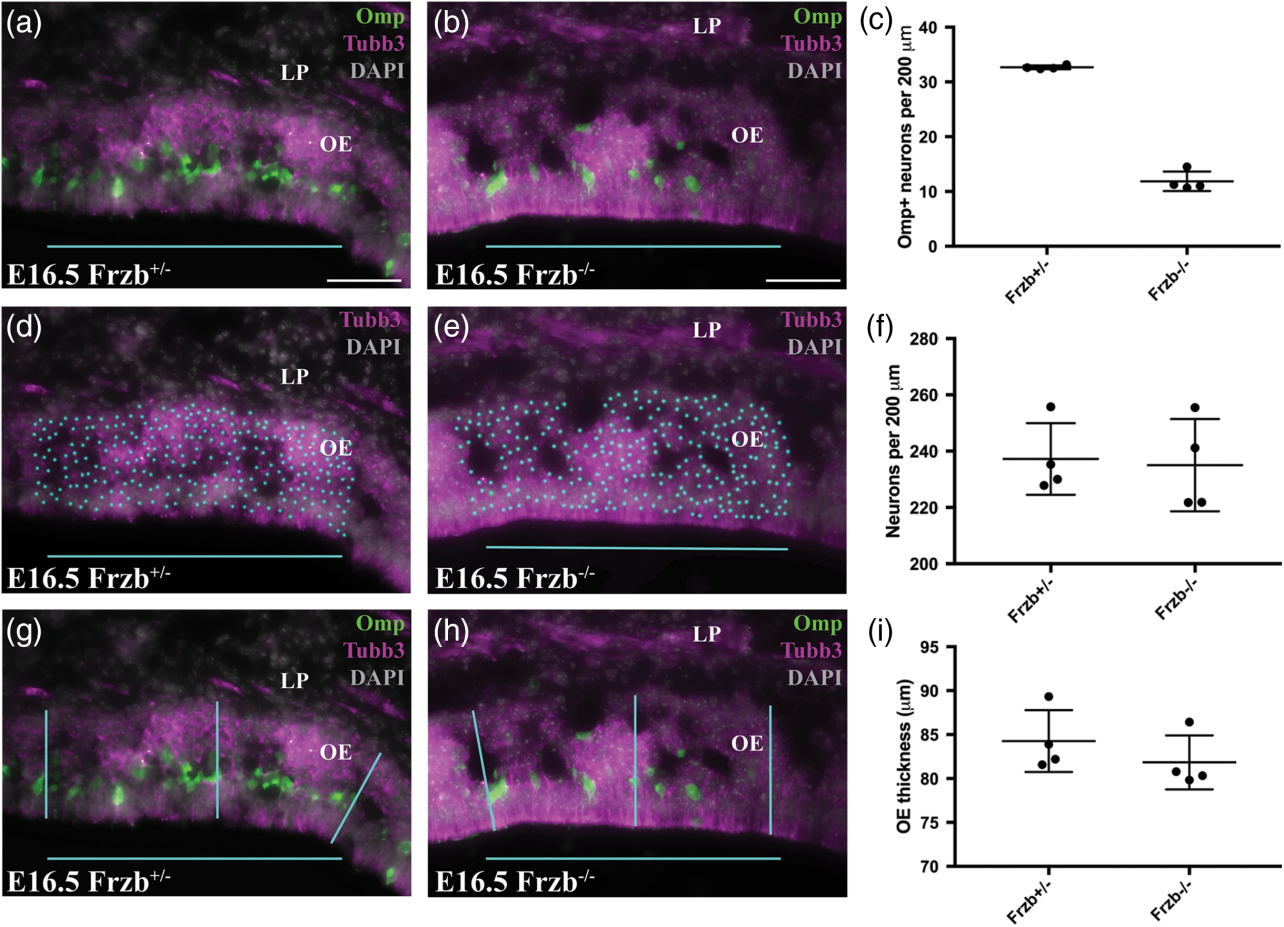
*Frzb* deletion disrupts olfactory receptor neuron maturation, not overall neuron number or the thickness of the olfactory epithelium. (a,b) Example coronal sections through a region of dorsal olfactory epithelium at E16.5, immunostained for the maturation marker Omp and the general neuronal/axonal marker Tubb3 and counter‐stained with DAPI, from a heterozygous *Frzb* ^*+/−*^ mouse embryo (a) and a *Frzb*‐null embryo (b). All Omp‐positive (mature) neurons (cyan dots) have been counted within the 200 μm region highlighted by the cyan bar. (c) Scatter plot showing that there is a significant difference between the mean number per embryo of Omp‐positive (mature) olfactory receptor neurons per 200 μm of epithelium for heterozygous *Frzb* ^*+/−*^ embryos (*n* = 4) versus *Frzb*‐null embryos (*n* = 4). Error bars show *SD*. (d,e) The same sections as in (a,b), showing Tubb3 and DAPI only, with all Tubb3‐positive neurons counted (cyan dots) within the 200 μm region highlighted by the cyan bar. (f) Scatter plot showing there is no significant difference between the mean number per embryo of olfactory receptor neurons per 200 μm of epithelium for heterozygous *Frzb* ^*+/−*^ embryos (*n* = 4) versus *Frzb*‐null embryos (*n* = 4). Error bars show *SD*. (g,h) The same sections as in (a,b), showing example measurements of the thickness of the olfactory epithelium at three points within the 200 μm region highlighted by the cyan bar. (i) Scatter plot showing there is no significant difference between the mean thickness per embryo of the olfactory epithelium for heterozygous *Frzb* ^*+/−*^ embryos (*n* = 4) versus *Frzb*‐null embryos (*n* = 4). Error bars show *SD*. LP, lamina propria; OE, olfactory epithelium. Scale bar: 50 μm [Color figure can be viewed at wileyonlinelibrary.com]

To test the hypothesis that *Frzb* deletion disrupts the maturation of olfactory receptor neurons, we compared the proportion of olfactory receptor neurons expressing the maturation marker Omp at E16.5 in heterozygous *Frzb* ^*+/−*^ embryos (*n* = 4) versus *Frzb*‐null embryos (*n* = 4) (Figure 6a–i). A 200 μm span of dorsal olfactory epithelium was selected on coronal sections (both left and right sides measured on each of 3–4 sections per embryo) or parasagittal sections (6 sections per embryo). Within the selected span of olfactory epithelium, we counted the number of Omp‐positive (mature) Tubb3‐positive neurons (Figure 6a–c) and the overall number of Tubb3‐positive neurons (Figure 6d–f), and measured the thickness of the olfactory epithelium at three different points (Figure 6g–i).

The mean number per embryo of Omp‐positive (mature) olfactory receptor neurons per 200 μm of epithelium (± *SD*) was 32.67 ± 0.35 for heterozygous *Frzb* ^*+/−*^ embryos (*n* = 4; Figure 6c), versus 11.85 ± 1.78 for *Frzb*‐null embryos (*n* = 4; Figure 6c). The *Frzb*‐null dataset did not pass the Shapiro–Wilk test for normality (*p* = .042); comparison of the datasets using the nonparametric Mann–Whitney (Wilcoxon rank sum) test showed that they were significantly different (*p* = .029).

In contrast, the number of olfactory receptor neurons per 200 μm of epithelium was not significantly different for heterozygous *Frzb* ^*+/−*^ embryos (mean per embryo ± *SD*: 237.2 ± 12.74; *n* = 4; Figure 6f) versus *Frzb*‐null embryos (235.1 ± 16.40; *n* = 4; Figure 6f) (unpaired two‐tailed Student's *t* test; *p* = .84). Similarly, *Frzb* deletion had no effect on the thickness of the olfactory epithelium, for which the mean per embryo (± *SD*) was 84.25 ± 3.52 μm for heterozygous *Frzb* ^*+/−*^ embryos (*n* = 4; Figure 6i), versus 81.84 ± 3.08 μm for *Frzb*‐null embryos (*n* = 4; Figure 6i). The *Frzb*‐null dataset did not pass the Shapiro–Wilk normality test (*p* = .035); comparison of the datasets using the Mann–Whitney test showed that they were not significantly different (*p* = .200).

Overall, these data show that *Frzb* deletion has no effect on either the overall number of olfactory receptor neurons or the thickness of the olfactory epithelium. In contrast, significantly fewer olfactory receptor neurons in *Frzb*‐null embryos express the maturation marker Omp. Given this, our results suggest that Frzb secreted by OECs abutting the olfactory bulb is required for normal olfactory axon targeting, hence for the maturation of olfactory receptor neurons.

### 3.5 *Frzb* deletion does not significantly affect GnRH neuron entry into the forebrain

**Figure 7:**
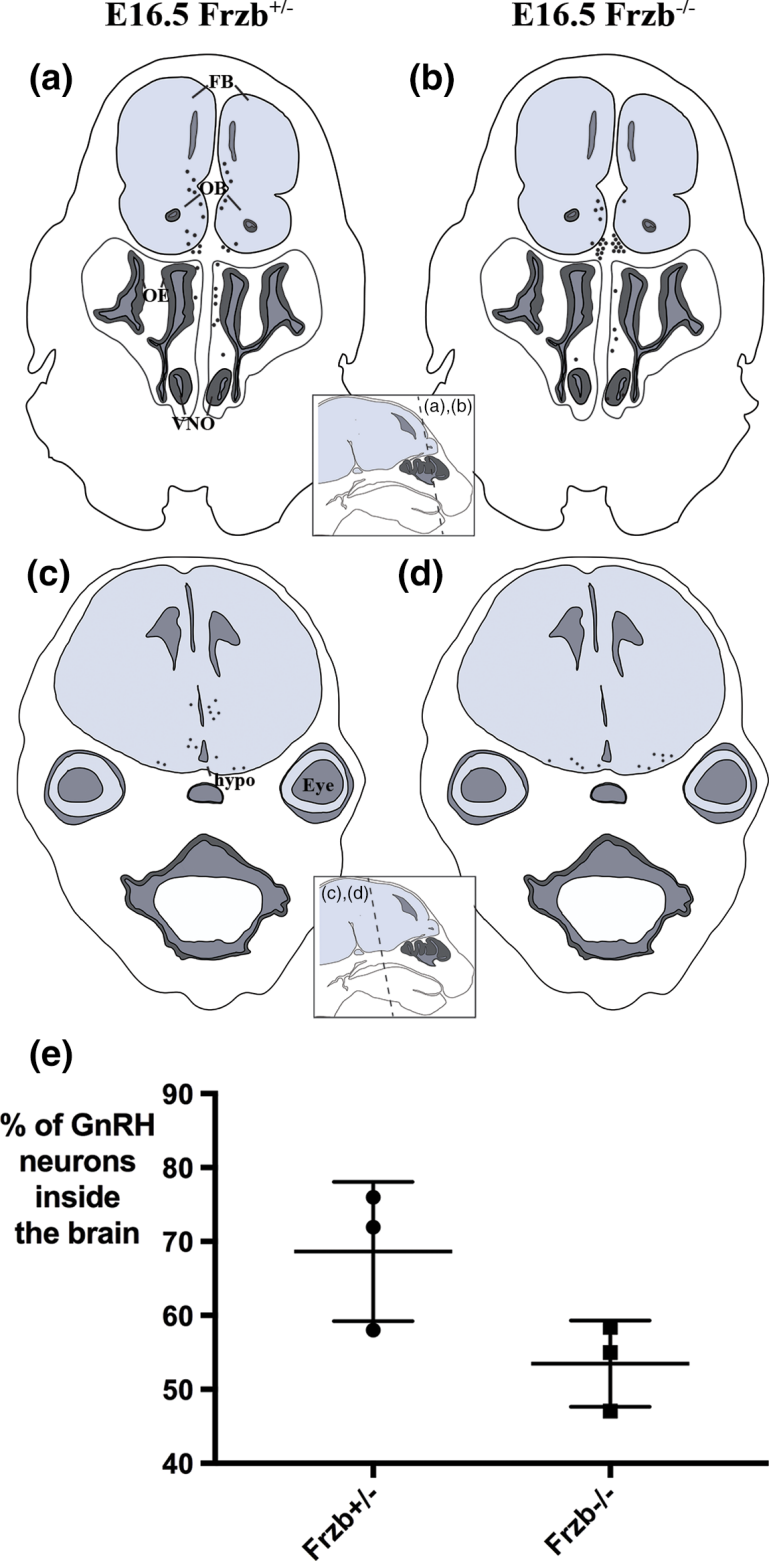
*Frzb* deletion does not significantly affect GnRH neuron entry into the forebrain. (a–d) Schematic representations of the distribution of GnRH neurons (black spots) at E16.5 on coronal sections at different rostrocaudal levels of a heterozygous *Frzb* ^*+/−*^ mouse embryo (a,b) and a *Frzb*‐null mouse embryo (c,d). Each dot represents a single GnRH neuron counted on one slide of a 10‐slide series. (e) Scatter plot showing the mean percentage of GnRH neurons found inside the brain for heterozygous *Frzb* ^*+/−*^ embryos (*n* = 3) versus *Frzb*‐null embryos (*n* = 3). Error bars show *SD*. FB, forebrain; hypo, hypothalamus; OB, olfactory bulb, OE, olfactory epithelium; VNO, vomeronasal organ [Color figure can be viewed at wileyonlinelibrary.com]

As described earlier, *Frzb* expression is first seen in OECs abutting the developing olfactory bulb at E12.5, when the first GnRH neurons enter the forebrain (Cariboni et al., 2007; Schwanzel‐Fukuda & Pfaff, 1989; Wray et al., 1989; Yoshida et al., 1995). GnRH neuron entry into the forebrain is disrupted in *Sox10*‐null embryos, in which normal OEC differentiation fails (Barraud et al., 2013; Pingault et al., 2013) and *Frzb* expression in the ONL is lost (Figure 3e–h^2^). We therefore tested whether Frzb secretion from OECs adjacent to the developing olfactory bulb might be important for GnRH neuron entry into the forebrain. We counted all GnRH neurons on sections of 3 heterozygous *Frzb* ^*+/−*^ embryos and 3 *Frzb*‐null embryos at E16.5, and determined the proportion inside the forebrain. At least 85 GnRH neurons were counted per embryo (mean ± *SD* of GnRH neurons counted per embryo: 142.5 ± 57.1; *n* = 6). Figure 7a–d shows schematic representations of the distribution of GnRH neurons on coronal sections at different rostrocaudal levels of a heterozygous *Frzb* ^*+/−*^ embryo (Figure 7a,b) and a *Frzb*‐null mouse embryo (Figure 7c,d). The mean percentage of all GnRH neurons counted (± *SD*) that were located inside the forebrain was 68.63 ± 9.42% for heterozygous *Frzb* ^*+/−*^ embryos (*n* = 3; Figure 7e) versus 53.48 ± 5.82% for *Frzb*‐null embryos (*n* = 3; Figure 7e). Comparison of the means using an unpaired two‐tailed Student's *t* test showed that they are not significantly different (*p* = .077). This contrasts with the fourfold reduction in GnRH neuron entry seen after *Sox10* deletion (Barraud et al., 2013; Pingault et al., 2013), which also results in the loss of *Frzb* expression adjacent to the olfactory bulbs (Figure 3e–h^2^). Taken together, this suggests that the loss of *Frzb* expression on the embryonic olfactory nerve does not significantly affect the entry of GnRH neurons into the forebrain.

## 4 DISCUSSION

Here, we report specific expression of the secreted Wnt inhibitor gene *Frzb* in OECs in the olfactory nerve layer, from E12.5 until at least E16.5 (the latest stage examined), when the strongest expression of *Frzb* is seen in OECs in the inner ONL, next to the olfactory bulb. Given that *Frzb* is important both for the innervation of the dorsal horn of the mouse spinal cord (John et al., 2012) and for the breakdown of the basement membrane during primary mouth formation in *Xenopus* (Dickinson & Sive, 2009), this led us to investigate whether Frzb secretion from this subpopulation of OECs might be one of the mechanisms underlying the defects in embryonic olfactory axon targeting and GnRH neuron entry into the forebrain seen in *Sox10*‐null embryos (Amaya et al., 2015; Barraud et al., 2013 ; Pingault et al., 2013), in which normal OEC differentiation fails (Barraud et al., 2013; Pingault et al., 2013). Indeed, we found that *Frzb* expression in developing OECs requires *Sox10*, and that *Frzb* deletion results in a significant decrease in olfactory receptor neuron maturation. This indirectly suggests a role for *Frzb* in olfactory axon targeting, as Omp expression correlates with synapse formation (Farbman & Margolis, 1980; Monti Graziadei et al., 1980). However, GnRH neuron entry into the forebrain is not significantly reduced. These results support roles for OEC‐secreted Frzb in embryonic olfactory axon targeting, but not in GnRH neuron entry into the forebrain.

### 4.1 *Frzb* deletion disrupts olfactory receptor neuron maturation, suggesting an olfactory axon targeting defect

Omp, a small cytosolic protein, is critical for the physiological activity of olfactory receptor neurons (Buiakova et al., 1996; Dibattista & Reisert, 2016; Ivic et al., 2000; Kwon, Koo, Zufall, Leinders‐Zufall, & Margolis, 2009; Lee, He, & Ma, 2011; Reisert, Yau, & Margolis, 2007) and has been used for decades as a marker of maturation. Omp is first expressed by a few olfactory receptor neurons at E14 in the mouse, correlating with the onset of synaptogenesis (Farbman & Margolis, 1980; Monti Graziadei et al., 1980). Omp is also upregulated by olfactory receptor neurons when embryonic olfactory epithelium (explanted prior to the onset of Omp expression) is co‐cultured in direct contact with presumptive olfactory bulb tissue of the same stage (Chuah & Farbman, 1983). We found that *Frzb*‐null mice display a significant reduction both in the volume of the Omp‐immunoreactive ONL and in the number of Omp‐expressing olfactory receptor neurons in the olfactory epithelium. This suggests that Frzb secretion from OECs abutting the embryonic olfactory bulb is important for olfactory axon targeting, thus indirectly affecting olfactory receptor neuron maturation. *Frzb* expression was previously reported in the mouse ONL from postnatal day 7 until young adulthood (Shimogori et al., 2004), suggesting that *Frzb* expression is maintained.

How could Frzb affect olfactory axon targeting? Frzb (also known as Sfrp3) is a member of the Sfrp family of secreted Wnt inhibitors, which have an N‐terminal cysteine‐rich domain with homology to the Wnt receptor Frizzled (Fz), and a C‐terminal Netrin‐related motif that is also found in the axon guidance protein Netrin and in tissue inhibitors of metalloproteinases (Cruciat & Niehrs, 2013). Sfrp1 promotes retinal ganglion cell neurite extension and acts as a chemotropic guidance cue for retinal growth cones via the Fz2 receptor (Esteve, Trousse, Rodríguez, & Bovolenta, 2003; Rodriguez et al., 2005). Sfrp1 and Sfrp2 bind to and reduce the activity of the metalloproteinase ADAM10, whose substrates include N‐cadherin and the neural cell adhesion molecule L1 (Esteve et al., 2011); the latter has important roles in axon guidance (Maness & Schachner, 2007). *Frzb* itself seems to be required in dorsal spinal neurons for their normal innervation by cutaneous afferents from the dorsal root ganglia, although the mechanism is unclear (John et al., 2012). Similarly, future experiments are needed to determine the mechanism by which Frzb, secreted by OECs at the edge of the olfactory bulb, affects olfactory axon targeting (and thus, indirectly, olfactory receptor neuron maturation). Loss of Frzb secretion from this subpopulation of OECs could therefore explain, at least in part, the olfactory axon targeting defects seen in *Sox10*‐null mice, in which normal OEC differentiation is defective (Barraud et al., 2013; Pingault et al., 2013).

### 4.2 GnRH neuron entry into the forebrain is not significantly impaired after *Frzb* deletion

The entry of the first GnRH neurons into the forebrain occurs at E12.5 (Cariboni et al., 2007; Schwanzel‐Fukuda & Pfaff, 1989; Wray et al., 1989; Yoshida et al., 1995), correlating with the onset of *Frzb* expression in OECs adjacent to the developing olfactory bulb. *Sox10* deletion results in the loss of *Frzb* expression on the olfactory nerve, and also in a fourfold reduction in the proportion of GnRH neurons that have entered the forebrain at E16.5 (Barraud et al., 2013). However, we did not see a significant reduction in GnRH neuron entry at E16.5 after *Frzb* deletion, relative to heterozygous *Frzb* ^*+/−*^ embryos. This suggests that the loss of *Frzb* after *Sox10* deletion does not contribute to the defect in GnRH neuron migration seen in *Sox10*‐null embryos (Barraud et al., 2013; Pingault et al., 2013).

### 4.3 The importance of OEC heterogeneity

The identification of *Frzb* expression in OECs in the developing ONL, most strongly in OECs in the inner ONL bordering the embryonic olfactory bulb (which also express *Npy*), is an example of the heterogeneity of OECs in vivo. OECs explanted into culture rapidly change their transcriptomic profile (see, for example, Franssen, De Bree, Essing, Ramón‐Cueto, & Verhaagen, 2008; Ulrich et al., 2014), but some molecular differences have been identified between different OEC subpopulations in vivo. For example, OECs on the olfactory nerve and in the outer ONL are immunoreactive for Ngfr (p75^NTR^) but not glial fibrillary acidic protein (Gfap) or neuropeptide tyrosine (Npy), while those in the inner ONL are Ngfr‐negative, G fap‐positive, and Npy‐positive (Au et al., 2002; Franceschini & Barnett, 1996; Ubink & Hokfelt, 2000). Furthermore, OECs co‐cultured with axons display different behaviors, depending on their location of origin within the olfactory system, which may reflect their normal functions in vivo (Ekberg, Amaya, Mackay‐Sim, & St John, 2012; Ekberg & St John, 2014). In the peripheral olfactory nerve, axons expressing a given odorant receptor are found in heterotypic bundles (i.e., bundled with axons expressing other odorant receptors); in the outer ONL, they are mostly defasciculated or in small fascicles, while in the inner ONL, they are found in larger, almost entirely homotypic axon bundles, before amalgamating into a glomerulus (Miller et al., 2010a; Treloar et al., 2002). Peripheral OECs (from the olfactory mucosa) promote greater neurite outgrowth from co‐cultured dorsal root ganglion neurons than do central OECs (from the olfactory bulb) (Roloff, Ziege, Baumgärtner, Wewetzer, & Bicker, 2013). Peripheral OECs consistently closely interact with and bundle co‐cultured olfactory axons, while central OECs (a mixed population from both the inner and outer ONL) have various effects on co‐cultured olfactory axons, including repulsion, adhesion, and crossover (Windus et al., 2010). These results are consistent with a role for OECs from the outer ONL in defasciculating axon bundles, and for OECs from the inner ONL in refasciculating and/or sorting axons expressing the same odorant receptor (Ekberg et al., 2012; Ekberg & St John, 2014). OECs show considerable potential for use in autologous transplantation therapies for nervous system repair (see, for example, Ekberg et al., 2012; Ekberg & St John, 2014; Granger, Blamires, Franklin, & Jeffery, 2012; Radtke & Kocsis, 2014; Roet & Verhaagen, 2014; Roloff et al., 2013; Tabakow et al., 2013; Watzlawick et al., 2016). Our results contribute to a greater understanding of the normal development and heterogeneity of OECs in vivo that may help inform these efforts.

## AUTHOR CONTRIBUTIONS

CVHB and CAR designed the study and wrote the article. CAR performed most of the experiments, analyzed the data, and prepared Figures 4, 5, 6, 7. SNP cloned mouse *Npy*, collected the data shown in Figures 1, 2, 3, and prepared these figures. JA and SB contributed genotyped *Frzb* mutant mouse embryos; CCS, MW, DPB, and EMS contributed genotyped *Sox10* ^*lacZ*^ mouse embryos. All authors read and commented on the article.
